# Inhibition of LCMR1 and ATG12 by demethylation-activated miR-570-3p is involved in the anti-metastasis effects of metformin on human osteosarcoma

**DOI:** 10.1038/s41419-018-0620-z

**Published:** 2018-05-23

**Authors:** Xing Bao, Libo Zhao, Hanfeng Guan, Feng Li

**Affiliations:** Department of Orthopedics, Tongji Hospital of Tongji Medical College, Huazhong University of Science and Technology, 1095#, Jiefang Ave, Wuhan, 430030 People’s Republic of China

## Abstract

Epidemiological studies have demonstrated that metformin could mitigate the progression of several tumors. Although it has been proved that metformin could cause demethylation of DNA and lead to up-regulation of some encoding genes and non-coding RNAs, there is little data about the effects of metformin on metastasis, and the interaction between metastasis and autophagy in human osteosarcoma cells. Here, we found miR-570-3p was significantly down-regulated in human metastatic osteosarcoma tissues but not in non-metastatic osteosarcoma tissues. Metformin attenuates the metastasis and autophagy in osteosarcoma. Interestingly, this autophagy favors osteosarcoma cells invasion. Moreover, reduction of metformin-induced inhibition of autophagy could reverse the invasion suppression in osteosarcoma. Mechanistically, metformin increases miR-570-3p by the demethylation of DNA, and the upregulation of miR-570-3p repressed the translation of its target, LCMR1 and ATG12. Our results, for the first time, presents evidence that the miR-570-3p-mediated suppression of LCMR1 and ATG12 is involved in the metformin-induced inhibition of metastasis in osteosarcoma cells.

## Introduction

Osteosarcoma (OS) is the most common primary malignant bone tumor, which is characterised by an awfully high aggressiveness with rapid development of distant metastasis^1–3^. Current practices for the treatment of osteosarcoma include wide-margin surgical resection and chemotherapy; nearly 30–40% of patients fail to respond to chemotherapy, typically due to the emergence of metastasis^4,5^. The mechanisms underlying metastasis remain vague. A further investigation of molecular markers that predict metastasis of osteosarcoma is urgent.

Increasing evidence reveals that microRNAs (miRNAs) play critical roles in regulating migration and invasion in tumor cells^6–8^. MiRNAs, which are small, noncoding RNA molecules of 20–25 nucleotides, have the capacity to suppress at the post-transcriptional and/or transcriptional level primarily by targeting the 3′-untranslated regions of mRNAs^9,10^. Aberrant expression of miRNAs have been found in many types of tumors^11–15^. Dysregulation of miRNAs can influence metastasis, chemosensitivity, and tumor cell growth and proliferation^16–20^. It has been reported that several mechanisms result to miRNA dysregulation, including aberrant DNA methylation of CpG islands^21,22^.

Metformin(Met), a relatively inexpensive and well tolerated oral anti-diabetic drug has raised extensive attention for its potential effects in tumor prevention and treatment^23,24^. An increasing number of studies have demonstrated varied mechanisms underlying the antitumor effect of metformin, with disparate mechanisms playing crucial parts in different tissues and in various tumors. It has been proved that metformin could cause demethylation of DNA and lead to up-regulation of some encoding genes and non-coding RNAs^25,26^. Many studies have shown that metformin could suppress the growth of tumor cells by leading to apoptosis and autophagy^27^.

Currently, there is little data about the effects of metformin on metastasis, and the interaction between metastasis and autophagy in human osteosarcoma cells. Understanding the interplay between metastasis and autophagy modulating by metformin may reveal new targets for tumor treatment. The influence of metformin on human osteosarcoma have not been sufficiently studied and, especially, the antitumor mechanisms of metformin in osteosarcoma have not been previously explored.

Here, we found that metformin weaken the migratory and invasive capacities of osteosarcoma cells in vitro and in vivo. Mechanistically, metformin increases miR-570-3p by the demethylation of DNA, and the increase of miR-570-3p repressed the translation of its target, lung cancer metastasis-related protein (LCMR1), which has an important role in tumor metastasis, and another target, autophagy-related gene 12 (ATG12), which is an autophagy marker that is significant for the resistance to apoptosis in many types of tumors.

## Result

### Metformin inhibits the migration and invasion of osteosarcoma cells

**Fig. 1:**
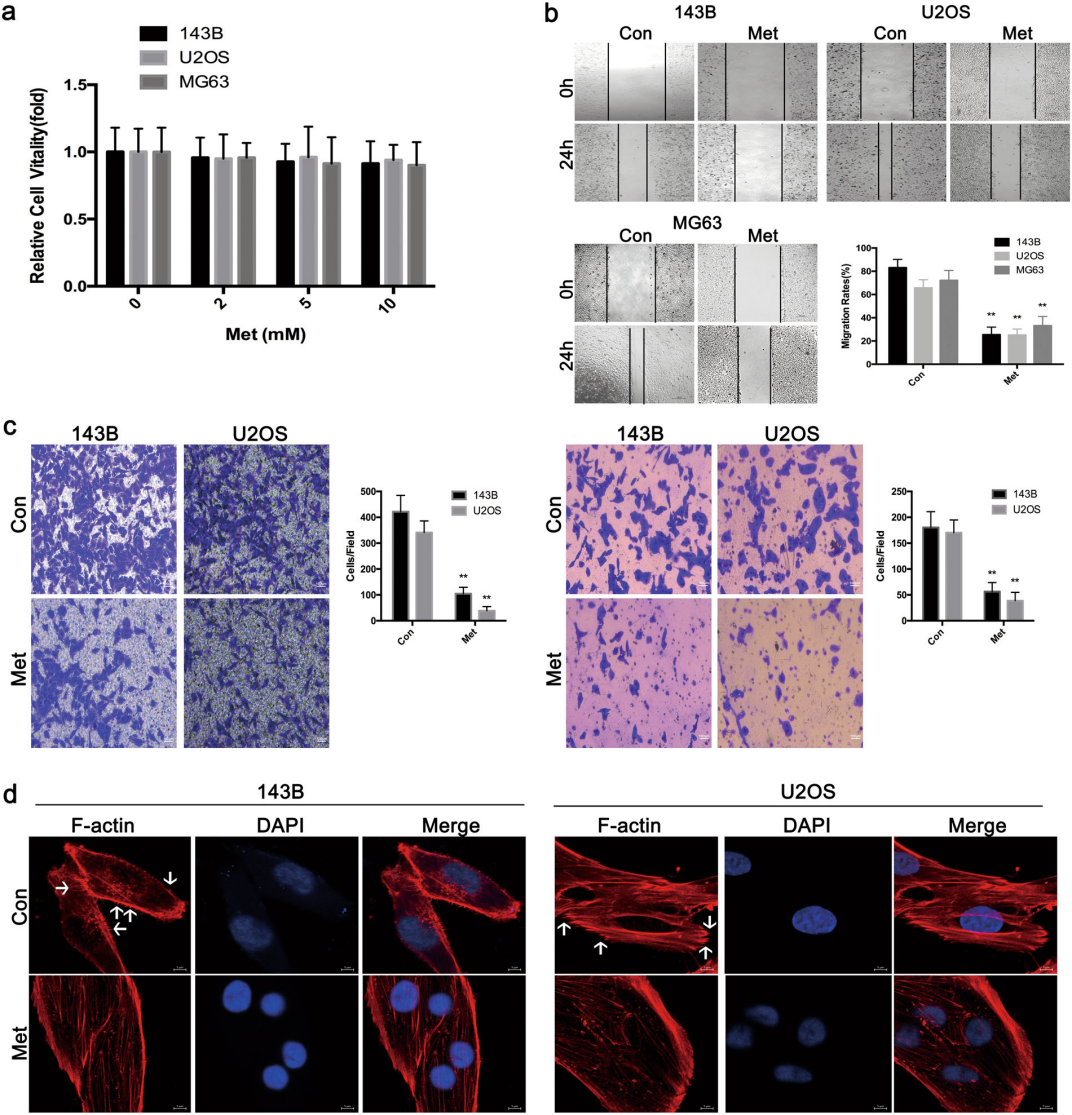
Metformin attenuates the migratory and invasive capacities of osteosarcoma cells. **a** CCK8 assays showed that metformin did not appreciably affect the vitality of MG63,U2OS or 143B cells at concentrations of 2, 5 or 10 mM (left). A similar result was detected in FCM experiments (right). **b** Metformin reduces the migration of osteosarcoma cells in wound-healing assays. Migration rates were calculated by the healing area/wound area after 24 h. **c** Metformin significantly suppresses the migration (left) and invasion (right) of osteosarcoma cells with transwell analysis. (*P < 0.05, **P < 0.01). **d** Cytoskeletal assay of 143B and U2OS cells was visualized by confocal microscopy. Representative images were shown. Cell nuclei were stained with DAPI. Scale bar represents 25 μm or 10 μm

Metformin did not markedly affect the vitality of MG63,U2OS or 143B cells at concentrations of 2, 5 or 10 mM (Fig. 1a). MG63,U2OS and 143B cells displayed high migratory and invasive capacities, although Metformin suppressed migration, as detected by wound healing assays, at a concentration of 10 mM (Fig. 1b). This concentration was selected as the maximum for further studies. Furthermore, as assessed with transwell assays, Metformin attenuated the invasion of osteosarcoma cells (Fig. 1c). These data reveal that metformin could efficaciously weaken the migration and invasion of human osteosarcoma cells.

Invasion of tumor cell is a trademark of metastasis, and that is a complicated process involving cytoskeletal rearrangement, lamellipodia forming, membrane ruffling and cell morphological alters. Cytoskeletal assay of 143B and U2OS cells was visualized by confocal microscopy. As demonstrated in Fig. 1d, conspicuous lamellipodial protrusions were formed in the submembranous area of control cells, but the reversed phenomenon was observed in metformin-treated cells, which displayed reconstructed cytoskeleton and well distributed F-actin in cells. This suggests that the treatment of Metformin could significantly affect cytoskeletal rearrangement in osteosarcoma cells.

### Metformin-induced inhibition of autophagy indirectly suppresses osteosarcoma cell invasion

Autophagy-associated cell death is another type of programmed cell death, which has emerged as an important regulator of the migration and invasion in different kinds of tumors^28^. In some metastatic tumors, high level of autophagy before treatment predicts invasiveness, low sensitivity to chemotherapy and poor survival^29^.

**Fig. 2:**
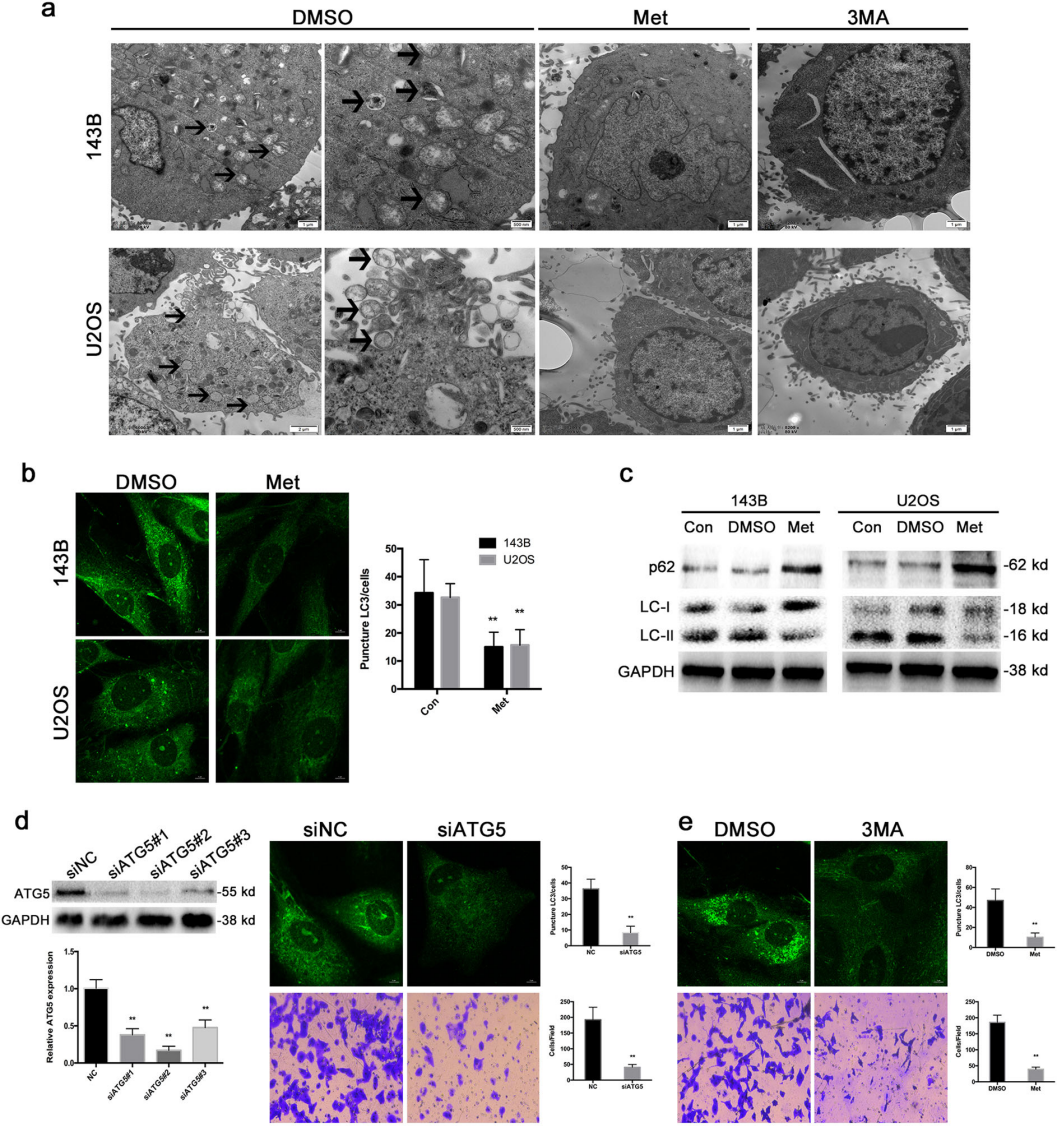
Metformin-induced inhibition of autophagy indirectly suppresses osteosarcoma cell invasion. **a** Representative TEM images depict the ultrastructures present during autophagy in 143B and U2OS cells treated with metformin or 3MA for 24 h. The images show autophagic vacuoles (arrows) observed in control cells. No or few autophagic vacuoles were observed in metformin and 3MA-treated cells. **b** Cells treated with metformin exhibited a punctate pattern of LC3-II fluorescence, showing a reduction of LC3-II in autophagosomes. **c** Western blot analysis was used to evaluate the expression of LC3 and p62. **d** Suppression of autophagy by knockdown of autophagy-related gene ATG5 could inhibit invasion of osteosarcoma cells. **e** Osteosarcoma cells treated with autophagy inhibitor 3MA also showed significantly inhibition of invasion

To figure out whether metformin is involved in autophagy, transmission electron microscopy (TEM) was carried out to observe the ultrastructures present during autophagy. Osteosarcoma cells treated with metformin and those treated with 3MA exhibited few autophagic vacuoles compared with typical autophagic vacuoles, and a distinct double membrane was present in the control group (Fig. 2a).

LC3 is a specific marker of autophagy initiation and is processed from LC3-I to LC3-II during autophagy. Then, the expression of LC3-II, as observed by immunofluorescence, can be used to track changes in autophagosome formation in osteosarcoma cells. As shown in Fig. 2b, cells treated with metformin exhibited few punctate pattern of LC3-II fluorescence, showing a reduction of LC3-II in autophagosomes. Decreased LC3-II expression and an accompanying increase in p62 expression were clearly detected by western blot (Fig. 2c).

Next, we wondered whether autophagy suppression was involved in the restraining effect of metformin on the migration and the invasion of osteosarcoma. Herein, we knockdown ATG5, a crucial gene for autophagy induction, in 143B cells with siRNA and an abrogation of cell invasion and migration was observed (Fig. 2d). In addition, we demonstrated that treatment with specific autophagy inhibitor 3-methyladenine (3MA) inhibited the invasion and migration of osteosarcoma cells (Fig. 2e), suggesting that both genetic and pharmacological inhibition of autophagy suppressed the invasive and migratory ability of human osteosarcoma cells.

### Metformin-mediated osteosarcoma cell invasion and autophagy by negatively regulating miR-570-3p expression in osteosarcoma

**Fig. 3:**
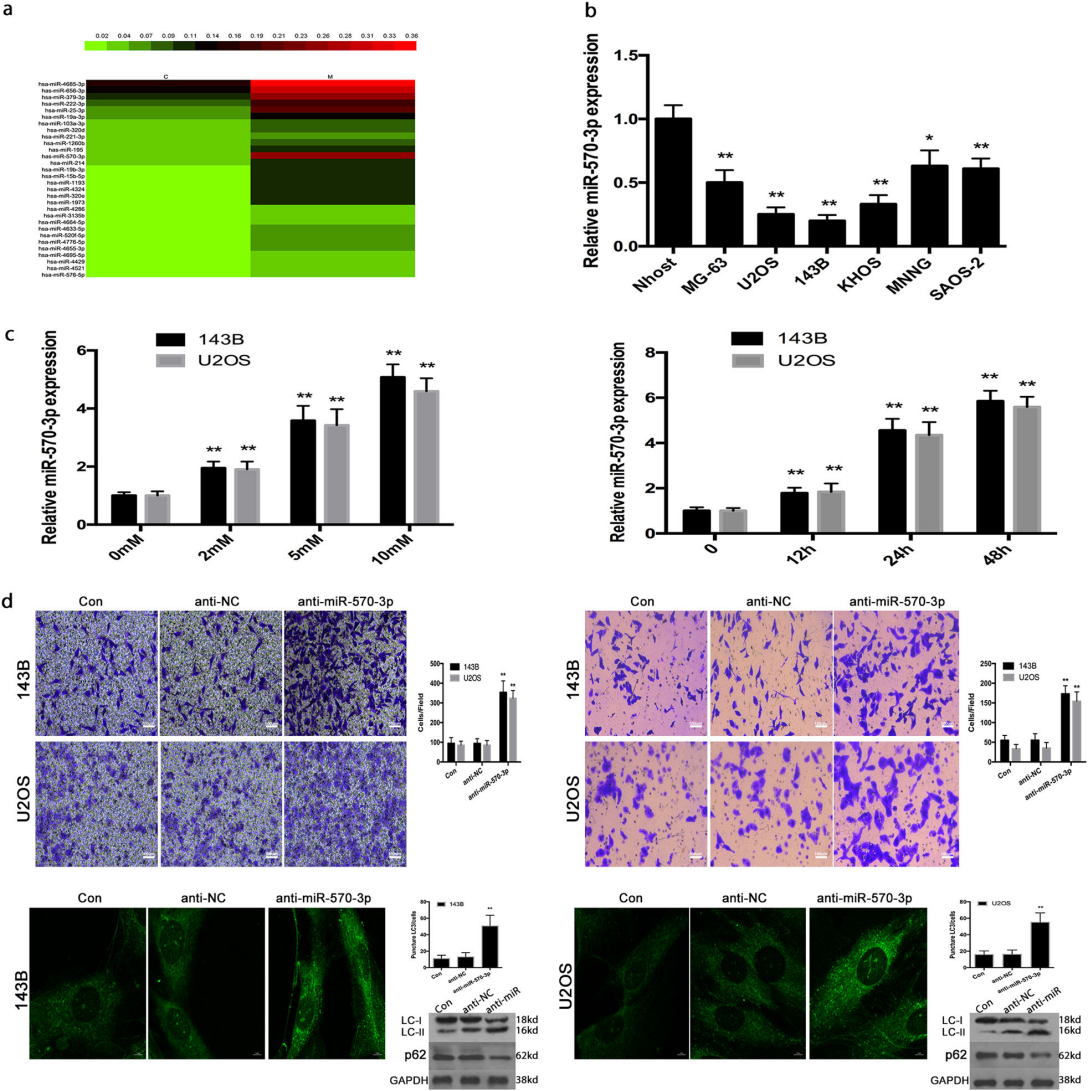
Metformin-mediated osteosarcoma cell invasion and autophagy by negatively regulating miR-570-3p expression in osteosarcoma. **a** Heat map of genes exhibiting significant induction (red) at 24 h after metformin treatment in 143B cells (expressed as a ratio to 143B cells treated with DMSO; fold change 42, *P*-value 0.05, data are log2 transformed). **b** The expression levels of miR-570-3p were examined in six osteosarcoma cell lines. **c** qRT-PCR demonstrated that miR-570-3p expression was markedly enhanced after metformin treatment in a dose- and time-dependent manner. **d** The knockdown of miR-570-3p reduced the metformin effects on invasion and autophagy in osteosarcoma cells. Data represent the mean ± S.D. (*n* = 3) in (**b**) and (**c**). **P < 0.01 by Student’s *t*-test

Recently, it is reported that metformin antitumor effects were proved to be connected, at least in part, with a regulation of microRNA expression^30^.To figure out the molecular mechanisms by which metformin affects osteosarcoma cell invasion and autophagy, we performed a miRNA microarray assay to screen for miRNAs regulated by metformin. Total RNA from 143B cells treated with metformin or DMSO was extracted and analyzed. We found that 23 miRNAs were upregulated, and among the changes in miRNA triggered by metformin, a marked increase in miR-570-3p was observed, which was the only one downregulated both in osteosarcoma tissues and cell lines (Fig. 3a).

Furthermore, miRNA mimics and inhibitors were used to explore the function of miR-570-3p. The expression levels of miR-570-3p were analyzed in six osteosarcoma cell lines, among which MG-63, KHOS, SAOS-2, MNNG cell lines showed significantly higher levels of miR-570-3p than U2OS and 143B cells (Fig. 3b). As demonstrated herein, in metformin-treated 143B and U2OS cells, there was increased expression of miR-570-3p (Fig. 3c). After 143B cells were transfected with anti-miR-570-3p or anti-con for 12 h, the tumor cells were exposed to 0.0 or 10 mM metformin for 48 h. Metformin attenuated the invasion and autophagy, as determined by transwell assays, in osteosarcoma cells, however, the knockdown of miR-570-3p decreased these effects in osteosarcoma cells (Fig. 3d).

These data suggest that miR-570-3p has a significant role in the metformin-induced inhibition of invasion and autophagy in human osteosarcoma.

### MiR-570-3p expression was down-regulated in human metastatic osteosarcoma tissues

**Fig. 4:**
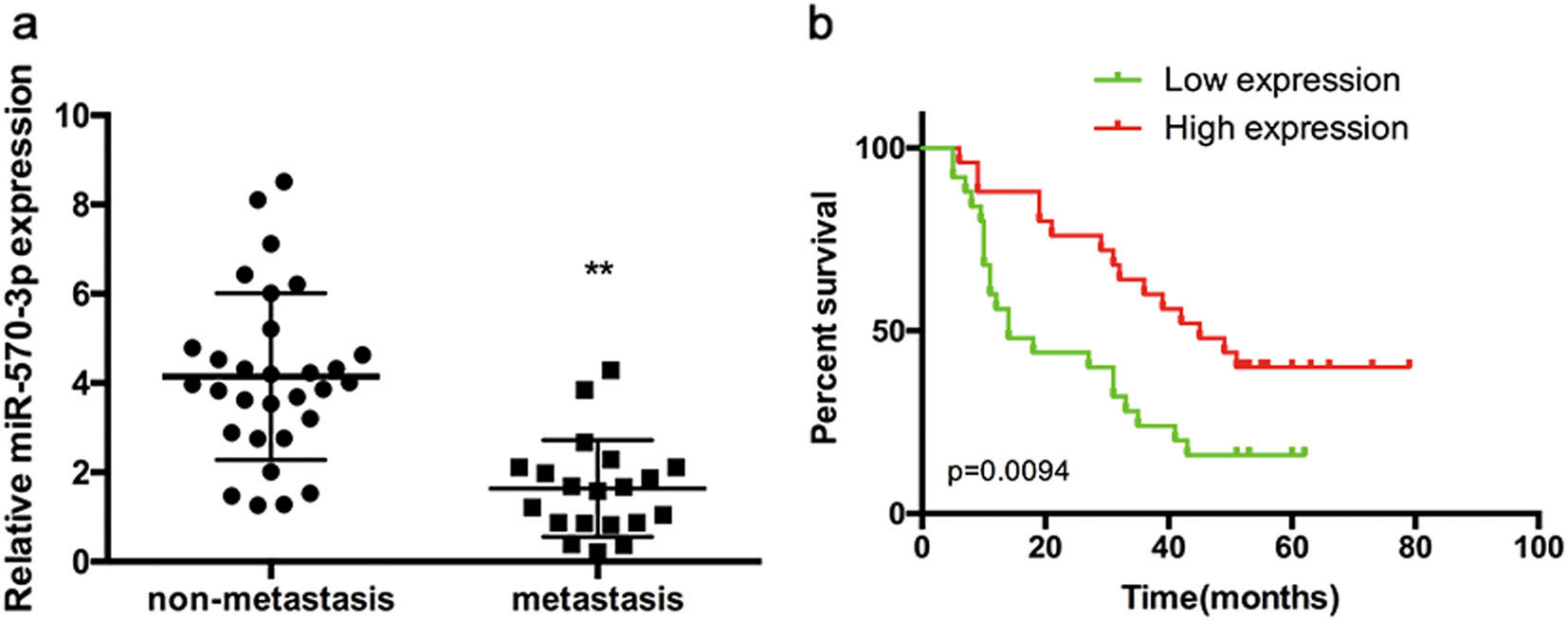
MiR-570-3p expression in human osteosarcoma tissues and its relation to patient survival. **a** The expression of miR-570-3p was significantly lower in metastatic osteosarcoma tissues than that in non-metastatic osteosarcoma tissues. **b** Overall survival of 45 osteosarcoma patients. The bars illustrated S.E.M. and the significant differences between samples were analyzed using Student’s *t*-test in B. All experiments were performed in three biological repeats

The expression level of miR-570-3p was quantified by qPCR in primary non-metastatic osteosarcoma tissues, metastatic osteosarcoma tissues and osteosarcoma cell lines. The expression level of miR-570-3p was markedly lower in metastatic osteosarcoma tissues than that in non-metastatic osteosarcoma tissues (Fig. 4a).

Next, we examined the correlation between miR-570-3p expression and osteosarcoma patient prognosis. Kaplan–Meier survival analysis displayed that the overall survival time of patients with low miR-570-3p expression was observably shorter than that of patients with high miR-570-3p expression (Fig. 4b).

### Metformin up-regulates the expression of miR-570-3p by the demethylation of DNA in 143B and U2OS cells

**Fig. 5:**
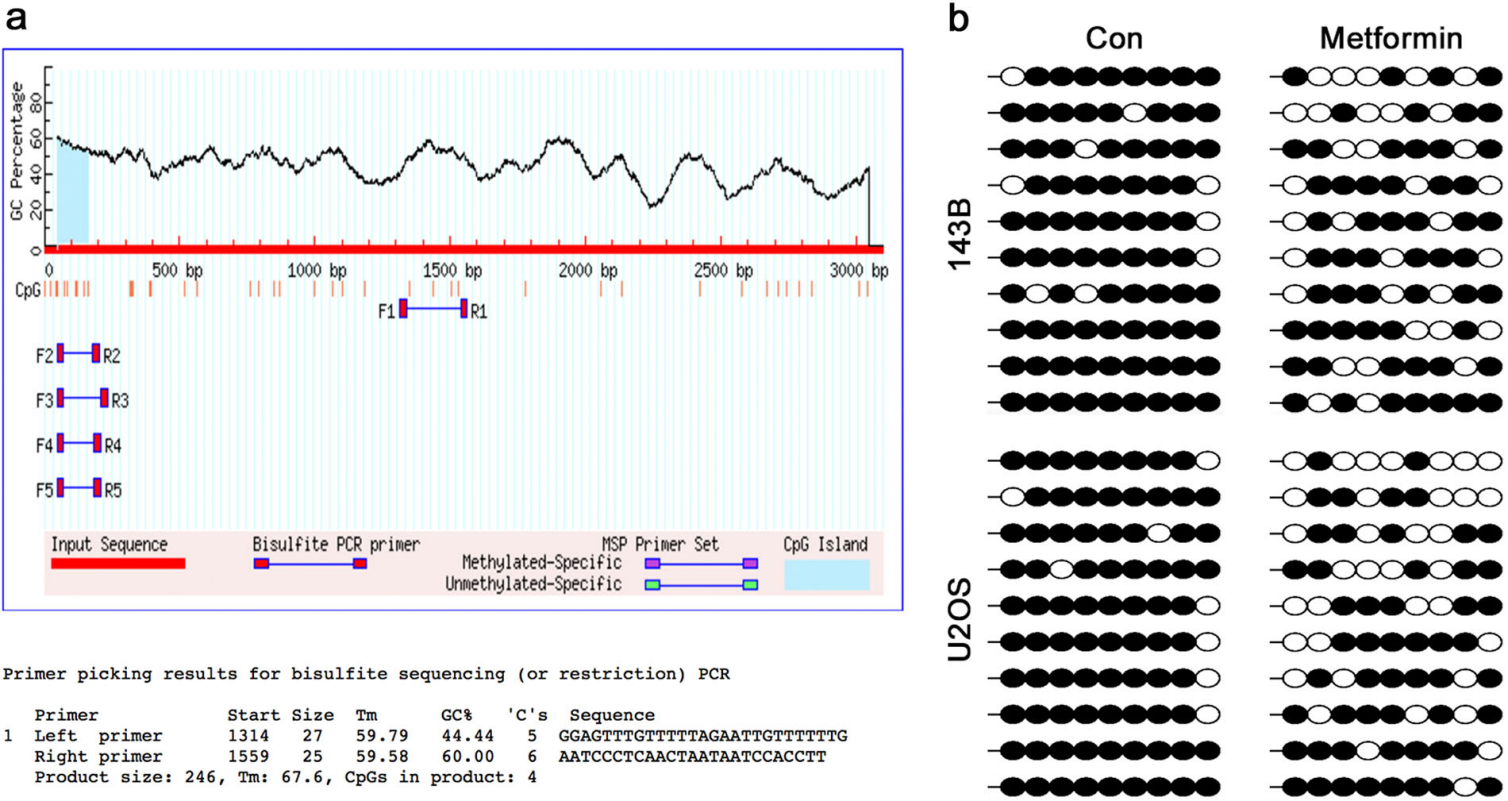
Metformin up-regulates the expression of miR-570-3p by the demethylation of DNA in 143B and U2OS cells. **a** The CpG islands of miR-570-3p promoter regions and primer used for DNA methylation study. **b** Metformin treatment in 143B and U2OS cells significantly decreased DNA methylation levels at the CpG islands of miR-570-3p promoter regions

Many studies have shown that metformin could cause demethylation of DNA and lead to up-regulation of some encoding genes and non-coding RNAs^25,26^. We hypothesized that metformin up-regulated the expression of miR-570-3p by demethylation. Bisulfate sequencing was used to analyze DNA methylation levels at the CpG islands of miR-570-3p promoter regions. The primer and CpG islands were shown in Fig. 5a. The results demonstrated that treatment with metformin in 143B and U2OS cells significantly decreased DNA methylation levels at the CpG islands of miR-570-3p promoter regions (Fig. 5b). Taken together, these data indicated that metformin-induced demethylation of miR-570-3p promoter may, at least in part, conduce to upregulation of miR-570-3p in osteosarcoma cells.

### LCMR1 and ATG12 are targets of miR-570-3p and initiators of metformin-induced metastasis inhibition and autophagy reduction

We explored the molecular mechanisms by which miR-570-3p affects metformin-mediated metastasis and autophagy suppression.

**Fig. 6:**
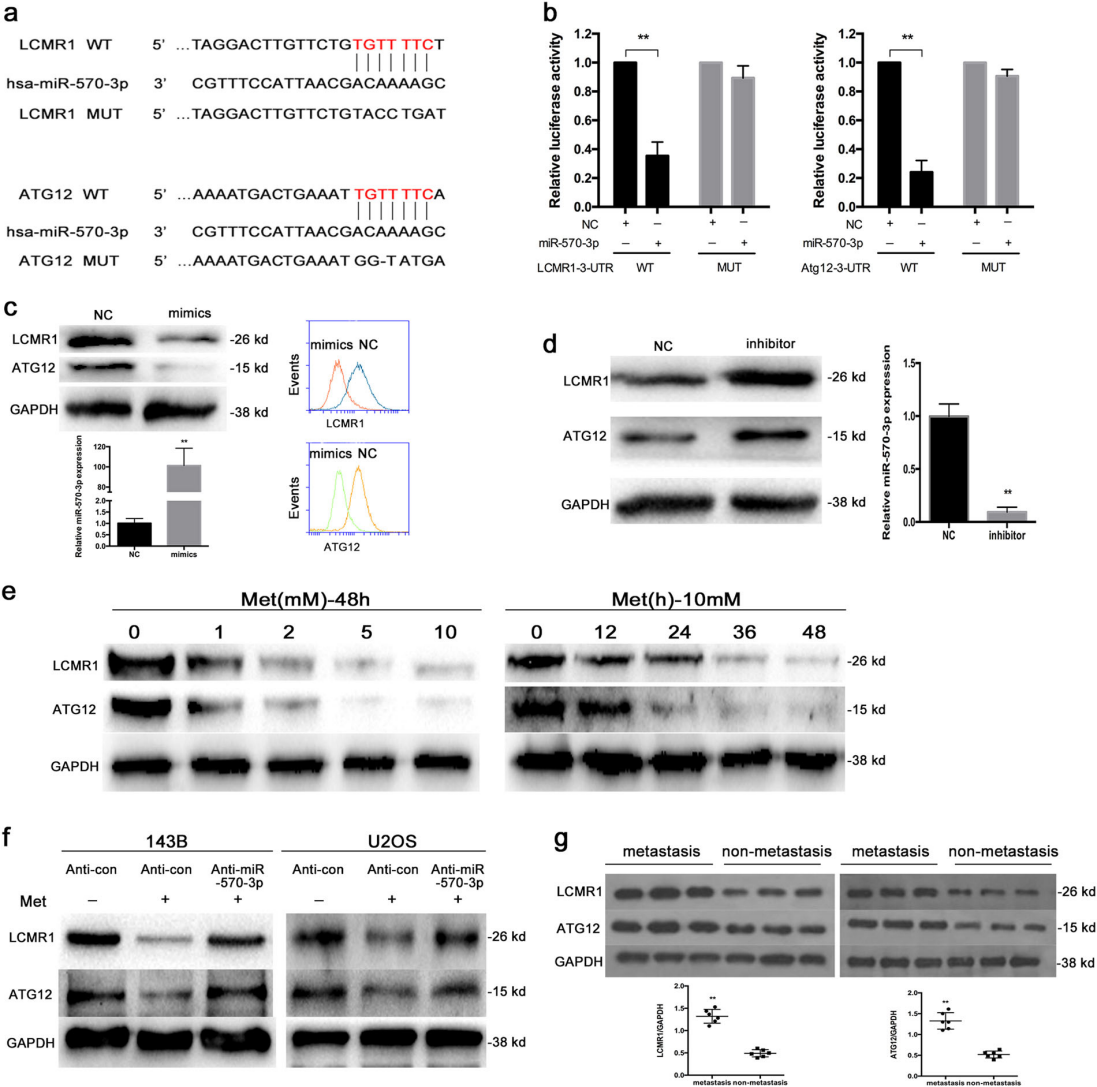
LCMR1 and Atg12 are targets of miR-570-3p and initiators of metformin-induced metastasis inhibition and autophagy reduction. **a** The LCMR1 and Atg12 3′UTR region containing the wild type or mutant binding site for miR-570-3p. **b** Dual-luciferase assays were performed in 143B cells after co-transfection with wild-type or mutant LCMR1 and Atg12 3′-UTR plasmids and with NC or miR-570-3p mimics. **c** Western blot and FCM demonstrated the significant downregulation of LCMR1 and Atg12 in miR-570-3p-overexpressing cells compared with NC cells. **d** Upregulation of LCMR1 and Atg12 in miR-570-3p knockdown cells. **e** Metformin inhibits LCMR1 and Atg12 in a time and dose-dependent manner. **f** Knockdown of miR-570-3p blocked the metformin-induced inactivation of LCMR1 and Atg12 in 143B and U2OS cells. **g** The expression of LCMR1 and Atg12 in metastatic and primary non-metastatic osteosarcoma tissues

As miRNAs function by targeting mRNAs, bioinformatics analyses using the target prediction tools miRNA, TargetScan and PicTar were used to identify potential binding sites in the 3′-UTRs of LCMR1 and ATG12 (Fig. 6a). We detected direct binding of miR-570-3p to the 3′-UTRs of LCMR1 and ATG12 using a dual-luciferase reporter assay (Fig. 6b) and observed significant down-regulation of LCMR1 and ATG12 in miR-570-3p-overexpressing 143B cells via western blot assay and FCM (Fig. 6c).

We also found that miR-570-3p knockdown up-regulated LCMR1 and ATG12 expression (Fig. 6d). In addition, we examined the effects of metformin on the expression of LCMR1 and ATG12 in osteosarcoma cells. As shown in Fig. 6e, LCMR1 and ATG12 were suppressed by metformin in a time and dose-dependent manner in 143B cells. Knockdown of miR-570-3p blocked the metformin-induced inactivation of LCMR1 and ATG12 in 143B and U2OS cells (Fig. 6f). Furthermore, over-expressing ATG12 and/or LCMR1 prevents effects of metformin and miR-570 on migration and invasion (Figure S1). Additionally, we analyzed the expression of LCMR1 and ATG12 in metastatic and primary non-metastatic osteosarcoma tissues. Our western blot assay suggested that LCMR1 and ATG12 expression was notably increased in metastatic osteosarcoma compared with the primary non-metastatic ones(Fig. 6g).

### Metformin up-regulates the expression of miR-570-3p and inhibits metastasis in vivo

**Fig. 7:**
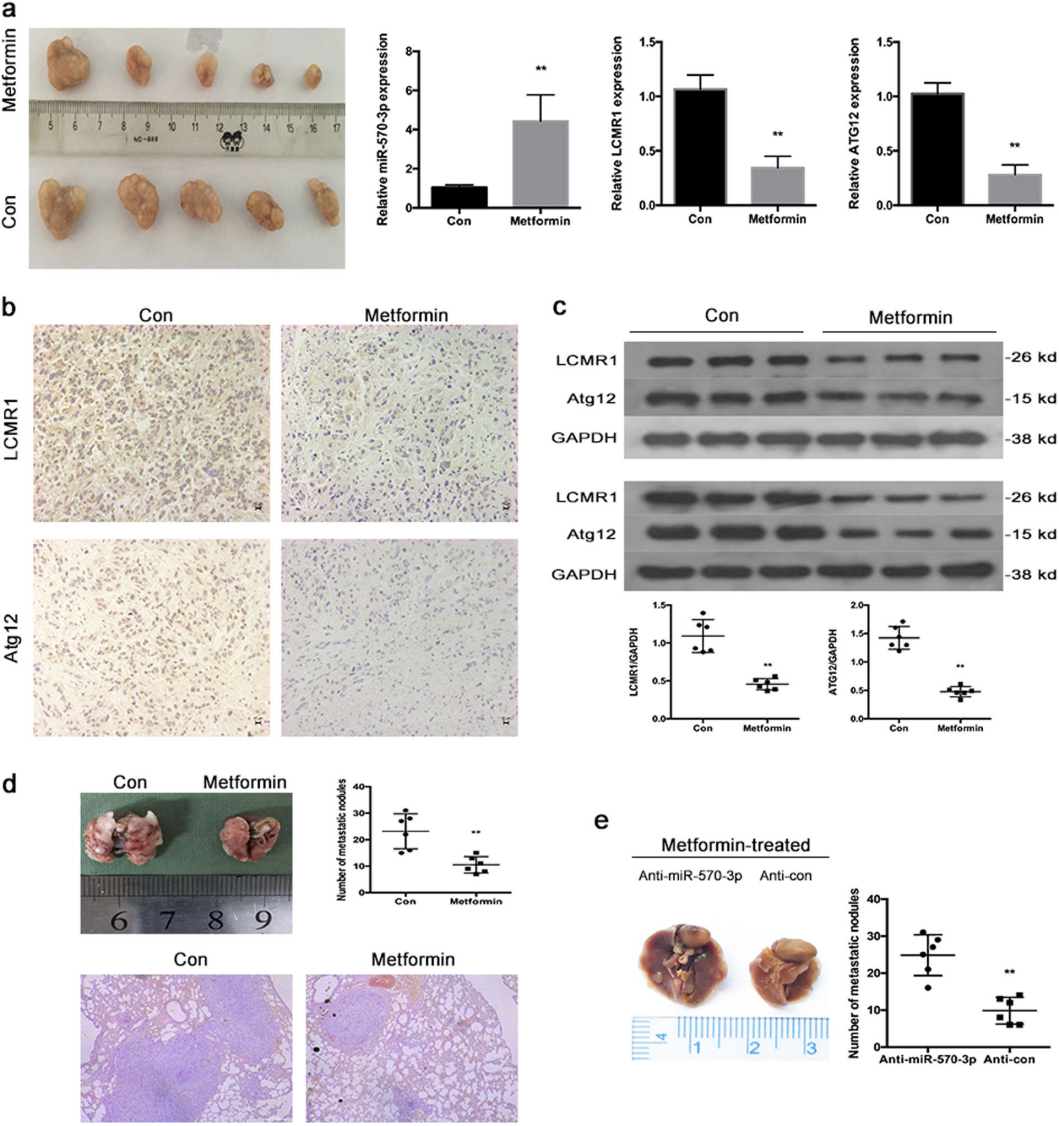
Metformin up-regulates the expression of miR-570-3p and inhibits metastasis in vivo. **a** Observation of tumors formed by 143B cells (left). qRT-PCR analyses of miR-570-3p, LCMR1 and Atg12 levels in xenografts (right) (mean ± SD, *n* = 3). **P < 0.01 compared with tumors treated with DMSO. **b** Immunohistochemistry analyses of LCMR1 and Atg12 levels. **c** Western blot analyses of LCMR1 and Atg12. **d** Number of metastatic nodules on the surface of the lungs of mice injected with DMSO or metformin is presented. Representative images and H&E staining of lungs on day 70 after the mice were injected with 143B cells (*n* = 6 per group). **e** Number of metastatic nodules on the surface of the lungs of mice formed by stable anti-miR-570-3p or anti-con 143B cells is presented

To further verify these in vitro findings, we used an in vivo xenograft model. After 143B cells were injected intravenously into the tail vein at 2 weeks, the mice were intraperitoneally administered metformin (0 or 250 mg/kg body weight) in 100 µl of sterile saline every second day for another 30 days. The metformin-treated group exhibited statistically notably fewer numbers of lung metastases than the control group. Hematoxylin and eosin (HE) staining showed that fewer lung metastatic nodes were detected in the group treated with metformin (Fig. 7d).

To validate the function of miR-570-3p in metformin-induced inhibition of metastasis, we established stable anti-con and anti-miR-570-3p 143B cell lines. Two weeks later, after the two stable cell lines were injected intravenously into the tail vein, the mice were treated as previously described. Compared to the anti-miR-570-3p group, fewer lung metastatic nodules were found in the anti-con group (Fig. 7e).The xenograft tumors were removed and evaluated by qRT-PCR, immunohistochemistry and WB (Fig. 7a–c).

## Discussion

Osteosarcoma is the most common primary malignant bone tumor. The cure rate of patients of osteosarcoma is still very low because of high aggressiveness with rapid development of distant metastasis.

Metformin is the most widely used drug for therapy of type II diabetes. Epidemiological studies have demonstrated that metformin could mitigate the progression of several tumors^24–27^. The earliest function of metformin is to reduce blood glucose levels through abating hepatic gluconeogenesis and increasing glucose uptake in the skeletal muscles^31^. It has been reported that metformin treatment results in activation of AMPK in vitro and in vivo^32–34^. Some studies have shown that metformin metabolic action leaded to a direct and broad regulation of some miRNAs that involved in the progression of tumors in vitro and in vivo^35,36^.

There is little known about the effects of metformin on metastasis, and the interaction between metastasis and autophagy in human osteosarcoma. Many investigations have implicated diverse mechanisms underlying the antitumor action of metformin, with different mechanisms playing important roles in different tissues and in different cancer types^37,38^. This difference may be related with tissue specificity. In the present study we found that a low concentration of metformin (10 mM) attenuates the metastasis and autophagy in osteosarcoma. Interestingly, this autophagy favors osteosarcoma cells invasion. Moreover, reduction of metformin-induced inhibition of autophagy could reverse the invasion suppression in osteosarcoma. Our data verified, for the first time, that autophagy regulates metastasis in osteosarcoma.

However, the mechanism about inhibition of metastasis and autophagy by metformin is obscure in osteosarcoma. MiRNAs are small non-coding RNAs that modulate gene expression by binding to the 3′-UTR of the target mRNA and suppressing translation or targeting the mRNA for degradation^12^. Almost all kinds of tumors, including osteosarcoma, display specific profile of aberrant miRNAs expression^39–48^.

To elucidate the molecular mechanism underlying metformin function, a miRNA microarray assay and bisulfite sequencing analysis (BSP) were performed. Herein, in metformin-treated human osteosarcoma cells, for the first time, we identified miR-570-3p as a tumor suppressor gene, which inhibited the metastasis and autophagy by targeting LCMR1(lung cancer metastasis-related protein), known as Med19 in human, which is crucial in the metastasis of various human tumors^49–51^, and another gene, autophagy-related gene 12 (ATG12), which is an autophagy marker that is significant for the development of many types of tumors.

MiR-570-3p has been reported to be dysregulated in various tumors^52,53^. According to our data, the expression level of miR-570-3p was significantly lower in metastatic osteosarcoma tissues than that in non-metastatic osteosarcoma tissues. We also investigated the methylation status of those sequences in metastatic and non-metastatic tumor tissues (Figure S2). We further validated that metformin increases the miR-570-3p expression by changing the DNA methylation level of miR-570-3p promoter regions.

Overall, this study reveals a promising therapeutics target in osteosarcoma and probably in other kinds of metastatic malignant tumors. We also propose a novel mechanism in which metformin demethylation-activate miR-570-3p and subsequently represses LCMR1 and ATG12 to inhibit metastasis and autophagy.In our study, we confirmed that the autophagy inhibition induced by metformin facilitated the suppression of invasion and metastasis, supporting the pro-metastatic role of autophagy in osteosarcoma. In addition, our findings suggest that miR-570-3p is a potential biomarker for predicting osteosarcoma metastasis and prognosis.

## Materials and methods

### Clinical specimens

Fifty osteosarcoma tissues were collected under the protocols approved by the ethics committee of Tongji Hospital. None of the patients received antitumor treatment before surgery. Informed consents (written in the light of the ethical guidelines) were obtained from all of the patients. The clinical characteristics of these patients were displayed in Table 1. Fresh tissues were stored in liquid nitrogen before RNA extraction. Clinical and histopathologic information was recorded through a retrospective review of patient records.

**Table 1:** The relationship between miR-570-3p expression and clinicopathological variables of osteosarcoma

Items	miR-570-3p		*P*-value
	Low	High	
All cases	25	25	
Age			0.3157
>18	8	11	
<18	17	14	
Gender			0.1816
Male	15	18	
Female	10	7	
Anatomical location			0.2352
Limb bone	13	19	
Axial bone	12	6	
Metastasis			0.0159
Present	13	7	
Absent	12	18	

### Cell culture and reagents

Normal human osteoblasts (NHOST) were obtained from Cambrex Bio Science and maintained in Osteoblast Growth Media (Lonza). The six human osteosarcoma cell lines MG63, U2OS, 143B, KHOS, MNNG, and SAOS2 cells were purchased from the American Type Culture Collection (ATCC). 143B, MNNG, and SAOS2 cells were cultured in Dulbecco’s modified Eagle’s medium (DMEM) (Hyclone, Logan, UT,USA) supplemented with 10% fetal calf serum (Gibco) at 37 °C in a humidified atmosphere with 5% CO_2_. KHOS and U2OS cells were cultured with RPMI 1640 medium (Invitrogen) supplemented with 10% fetal calf serum (Gibco). Cells lines were cultured at 37 °C with 5% CO_2_.

3MA (M9281) were purchased from Sigma Chemical Co. (St. Louis, MO,USA). These following antibodies were used in the experiments: anti-ATG5(9980), anti-LC3(4108), anti-p62(23214) and anti-GAPDH(2118) were from Cell Signaling Technology (Beverly, MA, USA). Anti-LCMR1 (ab179735), and anti-ATG12 (ab155589) was from Abcam (Cambridge, MA, USA).

### Transfection

SiATG5 and scrambled negative control siRNA (siNC) were synthesized in GenePharma (Suzhou, China). The sequences targeting ATG5 are listed in Supplementary Table S1. MiRNA mimics and inhibitors were purchased from RiboBio(Guangzhou, China). RNAs were transfected into tumor cells using Lipofectamine3000 (Invitrogen). MiR-570-3p and anti-miR-570-3p stably expressed osteosarcoma cells were infected with the lentivirus and selected with puromycin(1 μg/ml) for 4 weeks.

### CCK-8 assay

Cells were plated in 96-well plates at a density of 5000 cells in 100 μL medium per well 1 day before the experiment. The cell viability was examined by CCK-8 kit (Dojindo Laboratories, Kumamoto, Japan) according to the manufacturer’s instruction.

### Wound healing assay

A total of 2 × 10^5^ osteosarcoma cells were seeded into a 24-well plate. The tumor cells were grown to confluence 24 h later. An artificial wound was introduced with a P10 pipette tip in each well. The data of the wounded area were recorded at 0 h and 24 h with a microscope (Olympus Corp). The entire assay was repeated three times.

### Transwell assay

143B and U2OS cells were harvested, washed, suspended with RPMI1640 or DMEM medium, and seeded into the upper chambers of transwell inserts (8 μm pore size; Corning) with or without 10 mM Metformin in the migration assay. The upper chambers were coated with Matrigel (BD Bioscience, 354234) before the inoculation of the cancer cells and Metformin in the invasion assay.

The lower compartments were filled with RPMI1640 or DMEM medium supplemented with 5% FBS (fetal bovine serum). The cells in the upper chamber were removed with a swab after incubation for 12 h in the migration assay or 24 h in the invasion assay. The cells that migrated to the lower layer and attached to the membrane were stained with crystal violet and were counted in five fields per well under a microscope. The whole assay was repeated three times.

### Western blot analysis

Equal amounts of proteins collected from different kinds of cell lysates were loaded on 10–15% SDS-PAGE gels using a NuPAGE system (Invitrogen) and then transferred onto PVDF membranes as previously described^22^.

### Flow cytometry (FCM) experiments

Cells were fixed in 70% ethanol, digested with RNase A. Fluorescence-activated cell sorting was performed according to the manufacturer’s instructions. LCMR1 and ATG12-stained cells were quantified using flow cytometry as previously described^25^.

### Immunohistochemistry and immunofluorescence assay

IHC staining was performed as previously described^54^. Paraffin sections were reacted with rabbit polyclonal anti-LCMR1 and anti-ATG12 antibodies (1:100 dilution). Sections stained with non-immune rabbit serum (1:200 dilution) in phosphate-buffered saline (PBS) instead of primary antibody served as negative controls. Cells displaying positive staining were counted in at least 12 representative fields and the mean percentage of positive cells was calculated. Immunostaining was assessed by two independent pathologists blinded to clinical characteristics and outcomes.

For immunofluorescence assay of LC3, fixed cells were permeabilized with 0.1% Triton X-100 at room temperature for 15 min and incubated with anti-LC3 antibody overnight at 4 °C. Cells were washed 3 times with PBST (phosphate-buffered saline with Tween-20) and then incubated for 1 h with Cy3-conjugated goat anti-rabbit IgG at room temperature^27^. For immunofluorescence assay of cytoskeletal, cells (2 × 10^5^ cells per well) were adhered to coverslips in six-well plates. Cells were then fixed in 4% paraformaldehyde for 20 min and incubated in PBS with 0.1% Triton X-100 for 5 min. Coverslips were then moved to a piece of parafilm in a humid chamber to which 200 μl of 100 nM rhodamine phalloidin was added (Cytoskeleton Inc. Denver, USA). They were then incubated at room temperature shielded from light for 45 min. Nuclei were stained with PBS with 2 μg/ml DAPI for 4 min. The cells were then analyzed using confocal microscopy (FV10i, Olympus, Tokyo, Japan).

### Transmission electron microscopy (TEM)

After 48 h of metformin treatment, TEM assay was performed on cells. For TEM assay, cells were digested with 0.25% trypsin and suspended at a concentration of 1.0 × 10^6^ per ml and fixation was carried out at 4 °C for 6 h with 1.5% glutaraldehyde. Later, ultrathin sections (100 nm) were prepared, stained with uranyl acetate and lead citrate and examined under an electron transmission microscope (H-600; Hitachi, Tokyo, Japan)^54^.

### Quantitative RT-PCR (qRT-PCR)

The total RNA was extracted by Trizol reagent (Invitrogen). The cDNAs were synthesized using a RevertAidTM First Strand cDNA Synthesis kit (Fermentas, Vilnius, Lithuania), and real-time quantitative PCR was carried out using the SYBR-Green PCR Master Mix (Applied Biosystems, Foster City, CA, USA) on a 7900 Real-Time PCR System (Applied Biosystems). MiRNA qRT-PCR Primer were purchased from RiboBio. Other genes’ primer sequences are listed in Supplementary Table S1. U6 or GAPDH were used as endogenous controls. The reactions were performed in a 96-well optical plate(Applied Biosystems, Warrington, UK) at 94 °C for 2 min, followed by 38 cycles of 94 °C for 45 s, 56 °C for 45 s, and 72 °C for 40 s.

### MiRNA microarray assay

Total RNA from cells treated with metformin for 24 h was extracted using RNeasy mini kit (Qiagen, Venlo, The Netherlands), and reverse transcribed according to the manufacturer’s instructions (Fermentas, in CA). The miRNA microarray assay was performed by a commercial company (Phalanx Biotech Group, Hsinchu, Taiwan) using Human v7.1 miRNA OneArray platform that is designed to contain 100% of miRBase 21 database. Briefly, RNA was extracted using miRNA isolation kits (Qiagen®) according to the manufacturer’s protocol. RNA purified was quantified at OD 260 nm by an ND-1000 spectrophotometer (NanoDrop Technologies) and analyzed by the Bioanalyzer 2100 (Agilent Technologies, Santa Clara, CA, USA) with the RNA 6000 Nano LabChip kit. During the in vitro transcription process, 1 μg of total RNA was amplified by a low RNA input fluor linear amp kit (Agilent) and labeled with Cy3 (CyDye, PerkinElmer, Waltham, MA, USA). Using incubation with fragmentation buffer at 60 °C for 30 min, 1.65 μg of Cy3-labled cRNA was fragmented to an average size of about 50–100 nucleotides. Correspondingly fragmented labeled cRNA was then pooled and hybridized to SurePrint G3 ChIP/CH3 1 × 1 M array (Agilent) at 60 °C for 17 h. After washing and drying by nitrogen gun blowing, the microarrays were scanned with an Agilent microarray scanner at 535 nm for Cy3. Scanned images were analyzed by Agilent Feature Extraction, version 10.5. Image analysis and normalization software were used to quantify the signal and background intensity for each feature.

### Luciferase reporter assay

The LCMR1 and ATG12 3′-untranslated region (3′-UTR) containing the wild type or mutated miR-570-3p binding sequences were synthesized by Genescript (Nanjing, Jiangsu,China), and were cloned into the pmirGLO luciferase reporter vector (Promega, Madison, WI, USA). HEK293 cells were transfected with the wild type/mutant LCMR1 and ATG12 luciferase reporter vector and miR-570-3p mimic/miR-Control using Lipofectamine 3000. Firefly and Renilla luciferase activities were measured using the Dual-Luciferase Reporter Assay System (Promega). Results were expressed as the firefly luciferase activity normalized to Renilla luciferase activity.

### Bisulfite sequencing analysis (BSP)

The methylation levels of miR-570-3p promoter was analyzed by BSP. miR-570-3p DNA was extracted using a DNA kit (Qiagen 51306, Germany), and 2 μg of DNA was subjected to bisulfite conversion using an EpiTect Bisulfite Kit (59104, Qiagen, Germany) according to the manufacturer’s instructions. The transformed DNA was then PCR-amplified using the TaKaRa rTaq Kit (R001B, TaKaRa, Dalian, China). The PCR amplification products were sequenced by Invitrogen Corporation, Shanghai.

### Generation of xenografts

Six-week-old BALB/c female athymic nude mice (Vitalriver, Beijing, China) were subcutaneously injected in the right flank with osteosarcoma cells (2 × 10^6^ in 100 µl PBS). The volume of xenografts was measured every five days (tumor volume = (length × width^2^)/2). The mice were sacrificed 30 days later. Tumor samples were processed for IHC.

The tumor metastatic capacity of 143B cells (5 × 10^6^ cells) was detected following cell injection intravenously into the tail vein of mice. Four weeks later, the mice were divided into two groups at random and administered intraperitoneally with DMSO or metformin at a dose of 250 mg/kg every second day for 30 days (*n* = 6 per group); the mice were then sacrificed, and the number of lung metastatic nodules was counted. Metastatic lungs were fixed with 4% paraformaldehyde before dehydration and paraffin embedding. Paraffin sections were stained with hematoxylin and eosin according to standard protocols^55^.

### Statistical analysis

Data are expressed as the mean ± SEM of at least three independent experiments, and statistical evaluation was performed using one-way analysis of variance (ANOVA) or Student′s *t*-tests. Values of *P* < 0.05 were considered statistically significant.

## Electronic supplementary material

Table S1

Figure S1

Figure S2

Supplementary figure legends
