# Supplementary figures and images for: Inhibition of LCMR1 and ATG12 by demethylation-activated miR-570-3p is involved in the anti-metastasis effects of metformin on human osteosarcoma
Source: Cell Death Dis. 2018 May 23;9(6):611. doi: 10.1038/s41419-018-0620-z (PMC5966512; doi:10.1038/s41419-018-0620-z)

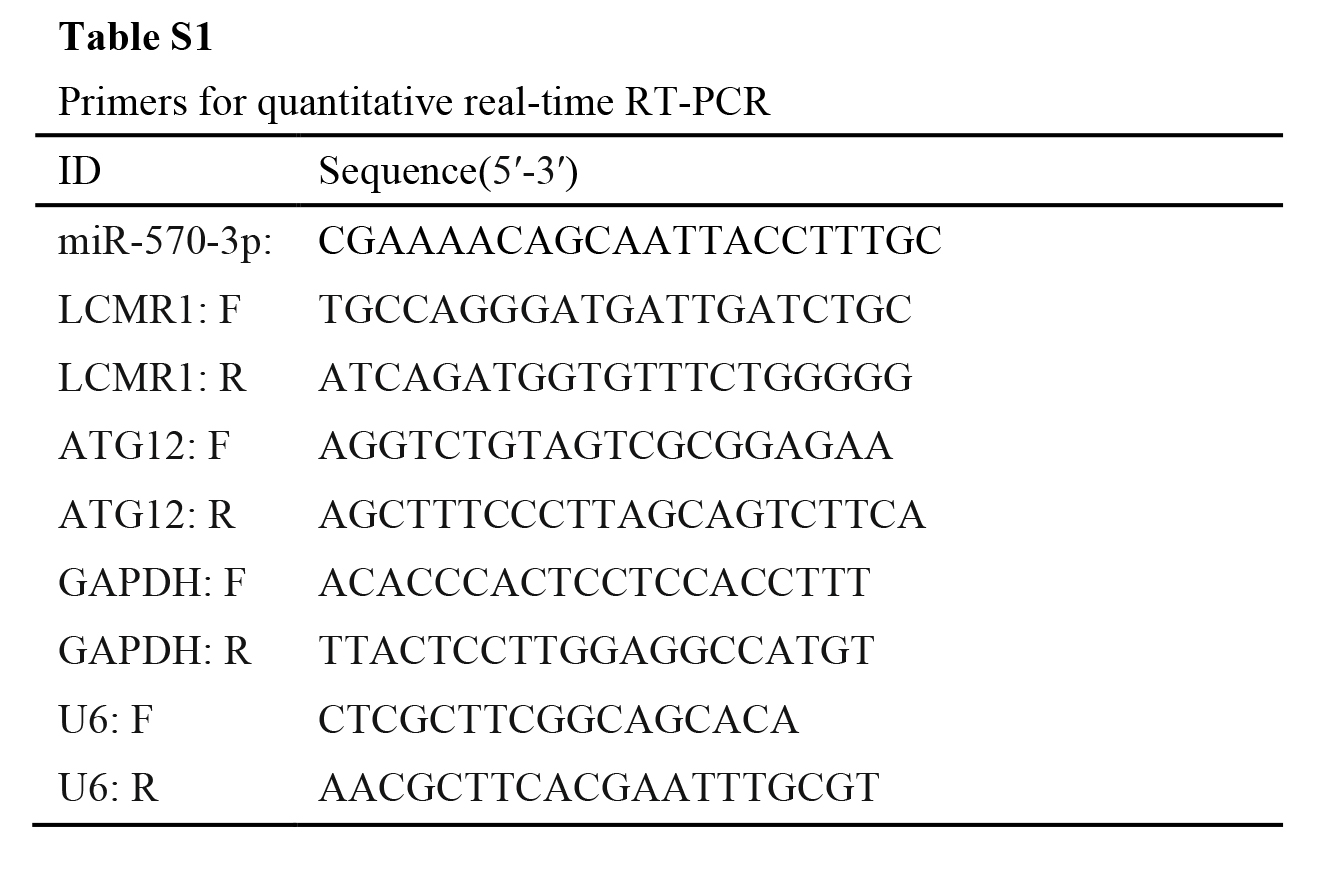

Supplement: Supplementary file 1 — Table S1 [file 41419_2018_620_MOESM1_ESM.jpg]

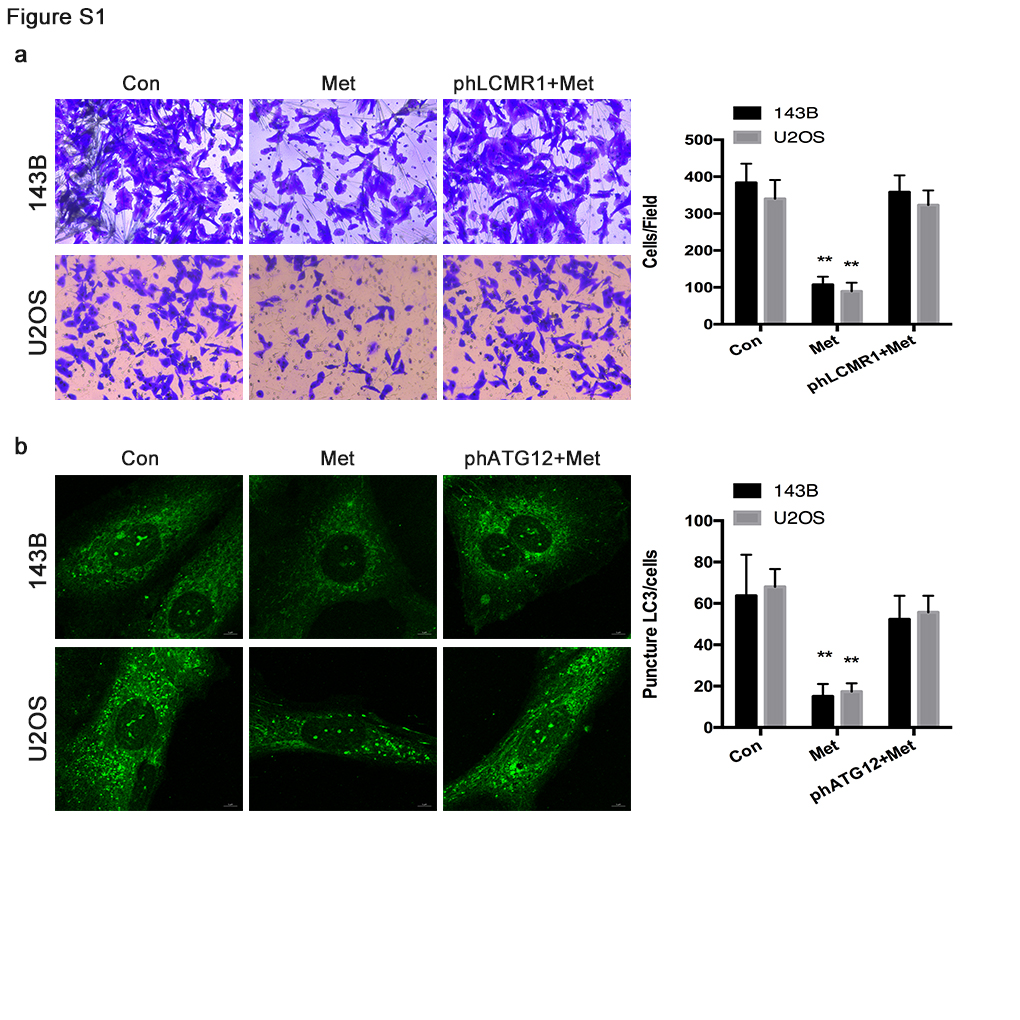

Supplement: Supplementary file 2 — Figure S1 [file 41419_2018_620_MOESM2_ESM.jpg]

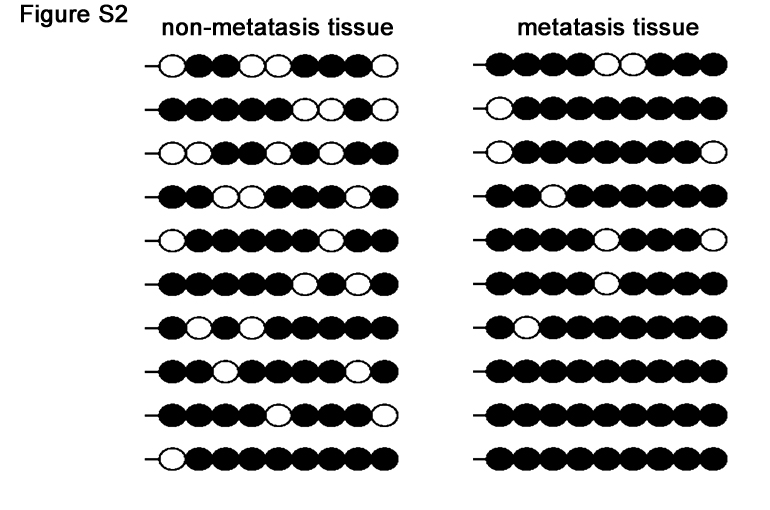

Supplement: Supplementary file 3 — Figure S2 [file 41419_2018_620_MOESM3_ESM.jpg]
