# Supplementary material for: Inhibition of LCMR1 and ATG12 by demethylation-activated miR-570-3p is involved in the anti-metastasis effects of metformin on human osteosarcoma
Source: Cell Death Dis. 2018 May 23;9(6):611. doi: 10.1038/s41419-018-0620-z (PMC5966512; doi:10.1038/s41419-018-0620-z)
Supplement: Supplementary file 4 — Supplementary figure legends [file 41419_2018_620_MOESM4_ESM.docx]

**SUPPLEMENTAL LEGENDS**

Supplemental Table S1. Primers for quantitative real-time RT-PCR

Figure S1

Over-expressing ATG12 and/or LCMR1 prevents effects of metformin and miR-570 on autophagy and invasion.

Figure S2

The methylation levels at the CpG islands of miR-570-3p promoter regions are higher in metastatic than that in non-metastatic tumor tissues.
